# A qualitative assessment of the pulmonary rehabilitation decision-making needs of patients living with COPD

**DOI:** 10.1038/s41533-022-00285-9

**Published:** 2022-06-29

**Authors:** A. C. Barradell, C. Bourne, B. Alkhathlan, M. Larkin, S. J. Singh

**Affiliations:** 1 Centre for Exercise & Rehabilitation Science (CERS), NIHR Leicester BRC (Respiratory), Glenfield Hospital, Leicester, UK; 2 Department of Respiratory Sciences, University of Leicester, University Road, Leicester, UK; 3 Applied Research Collaboration: East Midlands, College of Medicine, Biological Sciences and Psychology, Leicester General Hospital, Gwendoline Road, Leicester, UK; 4 Faculty of Health and Life Sciences, Coventry University, Priory Street, Coventry, UK; 5 School of Life and Health Sciences, Aston University, Aston Street, Birmingham, UK

**Keywords:** Chronic obstructive pulmonary disease, Rehabilitation, Patient education, Quality of life

## Abstract

Pulmonary rehabilitation (PR) is highly evidenced but underutilised in patients living with chronic obstructive pulmonary disease (COPD). A menu of centre and home-based programmes is available to facilitate uptake but is not routinely offered. An appraisal of the current PR referral approach compared to a menu-based approach was warranted to explore the decision-making needs of patients living with COPD when considering a referral to PR. Face-to-face or telephone, semi-structured interviews were conducted with patients diagnosed with COPD and referred to PR and referring HCPs. Interviews were audio-recorded, transcribed verbatim and analysed using the enhanced critical incident technique. 14 HCPs and 11 patients were interviewed (*n* = 25). Interview data generated 276 critical incidents which informed 28 categories (30 sub-categories). Five high-level themes captured patients’ decision-making needs for PR: Understanding COPD, understanding PR, perceived ability to access PR, a desire to accept PR, and supporting the offer. A menu-based approach would further support patients’ PR decision-making, however, insufficient knowledge of the programmes would limit its perceived feasibility and acceptability. The development of shared decision making interventions (e.g., a patient decision aid) to elicit patient-centred, meaningful discussions about the menu is suggested.

## Introduction

Chronic obstructive pulmonary disease (COPD) is a progressive respiratory condition characterised by chronic dyspnoea, cough, wheeze and excess sputum, punctuated by periods of acute exacerbation whereby symptoms worsen and rescue medication or hospitalisation is required^1^.

Optimal management of COPD includes completion of pulmonary rehabilitation (PR); a behaviour change intervention of progressive exercise and disease management education^2^ which improves dyspnoea, emotional functioning, self-efficacy and exercise capacity^3^. Traditionally, it is delivered as a supervised package of care over 6–8 weeks with two supervised sessions a week. However, despite its significant physical and psychological health benefits, uptake, attendance, and completion of the course is low^4^.

Patients who decline PR report a lack of transport options, perceived benefit, influence from healthcare professionals (HCPs), session times and disruption to their established routines as reasons not to attend^4–6^. In contrast, little evidence exists on the facilitators or improvements that can support patients’ decision-making and acceptance of PR. To date, the presence of social support, HCP support, motivation, self-efficacy and attributing value to PR has been cited as important^7^.

In response to these challenges and compounded by the onset of the Coronavirus Disease 2019 (Covid-19) pandemic, most PR services sought remote options to allow them to maintain a service. Most were developed in response to the pandemic context, but a few were developed and evaluated as options pre-pandemic (e.g. a standardised COPD self-management manual with remote support from HCPs (SPACE for COPD)^8,9^, an online programme involving exercise and education videos (my-PR)^10^). What is unclear is how these programmes are integrated into routine care and whether the choice of mode of delivery explicitly involves the patient. For example, SPACE for COPD is not offered to all referred patients, but often only suggested if the patient vocalises challenges in attending centre-based PR at the time of their PR initial assessment.

This approach is incongruent with shared decision-making; a core component of patient-centred care. The NICE guidelines describe shared decision-making as a process of HCPs and patients working together to make individualised choices about treatment and healthcare options^11^. This collaborative process ensures patients feel empowered, fully informed and can engage in key decisions about their health. Evidence shows that engagement in shared decision-making improves individuals treatment adherence, self-management and overall healthcare outcomes^12^.

To understand the role of shared decision-making within the offer of PR we sought to explore the PR decision-making needs of patients living with COPD to identify ways to support their PR decision-making and thereby their uptake to PR.

In this study, we conducted an exploratory qualitative analysis. Our research objectives were to understand:

- Patient and HCP perceptions of patients’ decision-making needs using the current PR approach: How do they perceive this approach with regard to its barriers, facilitators, and improvements?
- Patient and HCP perceptions of patients’ decision-making needs using a menu-based approach: How do they perceive this approach with regard to its barriers, facilitators, and improvements?

## Methods

### Study design

To capture a rich understanding of the factors influencing patients’ decision-making for PR, we utilised qualitative research methods. This allowed in-depth data collection specifically pertinent to individual participants^13^.

### Setting

We recruited participants from our local PR service. Patients were those referred to the service and HCPs were those who referred to the service from primary and secondary care sites.

### Participant selection

We used the proportionate allocation method of stratified sampling to recruit participants representative of our service. For patients, our sampling considered referral setting (e.g., inpatient, outpatient, GP setting) and residence (e.g. inner-city, urban). For HCPs, our sampling considered referral setting (e.g., primary care, secondary care) and site location (e.g. inner-city, urban).

Patients were eligible if they had a confirmed diagnosis of COPD (post bronchodilator FEV_1_/FVC ratio <70%), had received a PR referral and had not previously undertaken PR. This allowed reflection upon the decision-making processes for PR rather than prior experience of a PR programme. HCPs were eligible if they had been actively referring patients to the PR service for a minimum of 1 year. This ensured adequate experience to reflect upon.

We selected a sample size of 15 participants (e.g. 6–7 patients and 8–9 HCP), which is congruent with expert opinion on a minimum data set for qualitative research^14–16^. Throughout data collection, we assessed data saturation and continued recruitment until this was met, as per the analytic protocol, to ensure relevant contextual factors were captured.

### Data collection

Following participant consent, the first author, a Health Psychology Ph.D. student working in the field of respiratory research, collected baseline contextual data and conducted semi-structured interviews face to face or via telephone. Following the Covid-19 pandemic, we added additional contextual questions to the interview guides (Supplementary materials 1 and 2) and continued beyond the proposed sample size. Each interview was digitally recorded and transcribed verbatim. Transcripts were coded with an ID number and a ‘P’ to denote patient or ‘H’ to denote HCP. Data collection began in July 2019 and ended in October 2020. Interviews lasted between 19 and 45 min.

Interviews explored barriers, facilitators and improvements for the current PR approach (i.e. the offer of centre-based PR and possibly other options) compared to a newly proposed menu-based approach (i.e. the equal offer of: centre-based PR, home-based PR involving a self-guided manual and telephone support (SPACE for COPD)^8,9^, home-based PR involving a web-based manual and online and telephone support^10^ and for patients with fewer limitations: Active Lifestyles^17^ and Breathe Easy^18^). After exploration of the current approach, interviews were paused and participants read through a menu-based approach prompt (Supplementary material 3; telephone interview participants were sent a copy of the prompt in the post ahead of the interview). Following the opportunity to ask questions, the recording resumed exploring participant perceptions of this. A reflexive log^19^ was maintained prior to and throughout data collection to ensure data transparency.

### Data analysis

We conducted inductive data analysis using the Enhanced Critical Incident Technique^20^. This method identifies items, known as critical incidents, which make a significant positive (helping), negative (hindering), or future recommendation (wish list) contribution to the topic of interest. For example, and as previously highlighted, patients often cite a lack of transport options as a hinderance to their acceptance of PR. Here the hindering critical incident is ‘a lack of transport options’.

The first author extracted the helping, hindering and wish list critical incidents from the first transcript and grouped them thematically into categories. The remaining transcripts were analysed and critical incidents were placed into existing categories, or if incongruent, new categories were developed. The analysis continued iteratively until categories became specific and robust. To facilitate presentation of the results the categories were grouped into high-level themes.

The Enhanced Critical Incident Techniques’ nine credibility checks guided this process (Supplementary material 4). One check included the calculation of participation rates (i.e. the proportion of participants who contributed to each category), two included independent analysis by two additional authors (a Respiratory Ph.D. student and a Health Psychologist), one involved the verification of categories and quotes with participants and one involved the verification and saturation of categories with academic experts (an expert in the Enhanced Critical Incident Technique and an expert in Pulmonary Rehabilitation).

### Ethical approval

We received ethical approval by East Midlands—Leicester South Research Ethics Committee (REC: 17/EM/0156), the Health Research Authority and the research site. The trial is registered on Clinical Trials.gov (Identifier: NCT04990180). Participants provided written informed consent.

### Reporting summary

Further information on research design is available in the Nature Research Reporting Summary linked to this article.

## Results

Data exhaustion was reached at 25 interviews (14 HCPs and 11 patients). Participant characteristics are displayed in Table 1.

**Table 1:** Participant demographics.

HCPs	COPD patients
*N* = 14(56)	*N* = 11(44)
Gender (% female): 10(71)	Gender (female): 4(36)
Age: 42.4(26–57)	Age: 66.9(37–86)
Professions	Age at diagnosis: 62.2(37–83)
Nurse: 7(50)	Ethnicity
Specialist COPD Nurse: 2(14)	White British: 11(100)
Physiotherapist: 1(7)	No. of years diagnosed: 4.7(0–15)
General practitioner: 3(21)	
UHL Doctor: 1(7)	
Time in current job role: 6.2(1–20)	
Site location	Residence
Inner city: 9(64)	Inner city: 5(45)
Urban: 5(36)	Urban: 6 (55)
Referral setting	Referral site
Primary care: 8(57)	Inpatient: 2(18)
Secondary care: 6(43)	Outpatient: 6(55)
	GP practice: 3(27)

*N*(%) or mean(range).

**Fig. 1:**
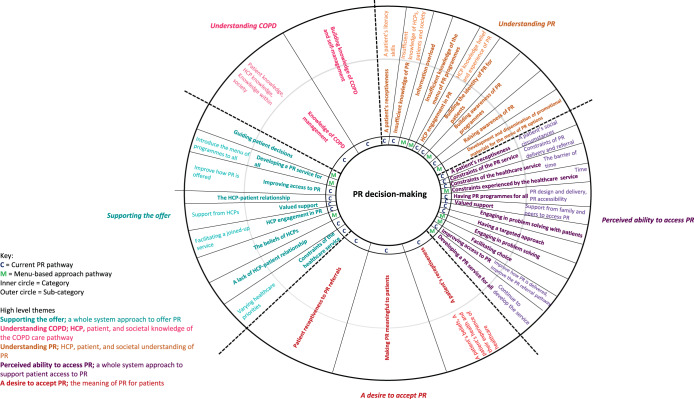
A thematic map of the generated categories and sub-categories, displayed by high-level theme.

We identified 276 critical incidents which generated 28 categories (30 sub-categories). Five high-level themes illustrated the helping, hindering and wish list critical incidents which influenced patients’ PR decision-making; Understanding PR, perceived ability to access PR, a desire to accept PR, supporting the offer, and understanding COPD (Fig. 1). The high-level themes are presented textually incorporating the relevant categories/sub-categories for the analysis of the current PR approach and the menu-based approach (where relevant). We use the term ‘participants’ when both HCPs and patients verify a category/sub-category. If the category/sub-category is unique to HCPs or patients, these terms are used instead.

### Understanding COPD

For the current approach, insufficient ‘Patient knowledge’ and ‘Knowledge within society’ were identified as barriers to positive COPD decision-making and self-management (Table 2). Participants described limited information provision or access to unofficial information online as culprits which could result in delayed decision-making regarding healthcare access, social support access, and treatment initiation.

**Table 2:** Understanding COPD.

Frame of reference (current PR/menu-based approach pathway) and Item type	Participation rate (%)	Category	Sub-category
Current; hindering	28	Knowledge of COPD management	*Patient knowledge* AB05P: “It’s got worse since March. When I had that first [exacerbation], I’d left it because I didn’t realise that you could get the steroids for it or anything.” *HCP knowledge* AB12H: “I asked [my colleagues] to put their hands up—I asked them the question Would you prefer to have a heart attack or infective exacerbation of COPD?’ And loads of people said infective exacerbation of COPD, and these are people that treat patients with COPD and heart attack all the time. And they didn’t even know that you’ve got a higher mortality risk with an infective exacerbation so I think as [healthcare] professionals we don’t know therefore the patients don’t know.” *Knowledge within society* AB08P: “…when you tell somebody that you’ve got COPD and they say ‘What do you mean?’ ‘Well you’ve heard of asthma, it’s the next bit up.’ ‘Eh!’ Unless they’re medical people other people have never heard of it. Nor had I til I got it, but there you go.”
Current; Wish list	36	*Building knowledge of COPD and self-management* AB01H: “I think anyone who is given that diagnosis should have to go through a programme and I know they perhaps do not need an exercise programme at that point but it’s almost like they need something like an education programme.”	

Additionally, insufficient ‘HCP knowledge’ was highlighted. Participants discussed the complexity of COPD management and felt without training updates, knowledge could decline, particularly amongst HCPs who treat patients with varying conditions. Consequently, this would result in insufficient understanding of COPD amongst patients too.

‘Building knowledge of COPD and self-management’ amongst HCPs, patients and society was proposed to facilitate healthcare decision-making with patients and thereby positive COPD management. Participants advocated for standardised provision of patient education to those newly diagnosed to promote positive self-management (e.g., the provision of PR education sessions to all newly diagnosed patients). Furthermore, the provision of top-up training for HCPs and formalising primary care respiratory leads were recommended too.

### Understanding PR

For the current approach ‘A patient’s literacy skills’ were felt to influence their understanding of PR and thereby their PR decision-making, particularly if offered home-based programmes where materials are provided with remote supervision (Table 3). Participants expressed, that whilst there is a trend for increasing computer and health literacy within society, within the COPD population this is limited and therefore the format of home-based programmes would not be comprehensible for a subset of patients.

**Table 3:** Understanding PR.

Frame of reference (current PR/menu-based approach pathway) and Item type	Participation rate (%)	Category	Sub-category
Current; Hindering	96	A patient’s receptiveness	*A patient’s literacy skills* AB02P: “You see to read a manual for me it would send me to sleep. I would be far better to talk to somebody like you and even if it’s in a group environment like sitting at a desk and just listening to somebody give a lecture. I would take more interest and I’d keep more awake doing that than I would be trying to read for myself. And probably not understanding what I was reading anyway.”
Current; Hindering	76	Insufficient knowledge of PR	*Insufficient knowledge of HCPs* AB17H: “I think probably a lot of people, me included, just wing it and you’ll speak to your boss because that’s what tends to happen in trainee clinics, ‘So I’ve seen this patient’ and you go through everything and then your boss will say ‘Refer them to pulmonary rehab’ and then you think ‘They’re gonna ask me about it, I dunno what that is’ so you Google it and you get a very, very basic superficial understanding of what it is enough to just say this is what it is.” *Insufficient knowledge within society* AB01H: “I think we take it for granted that everybody knows that [PR] exists… unless you’re in respiratory I don’t think it’s a common theme really.”
Menu; Hindering	44	*Information overload* AB10P: “Well having all this choice, I really don’t know what’s the best. I mean, I’d sooner somebody tell me ‘You’re doing that…’ That’s cos I really don’t understand my problem.”	
Menu; Hindering	36	*Insufficient knowledge of the menu of PR programmes* AB02P: “I’m not fond of information overload, but I’m equally not fond of not knowing at all. Therefore something in between is very useful and I’m finding this conversation very useful from that point of view.”	
Current; Helping	48	HCP engagement in PR	*HCP knowledge, belief and experience of PR* AB11H: “I think if [healthcare professionals] actually take part in a session, particularly if you can see patients that you’ve perhaps referred in, you actually get a much better flavour of what it is so that when you’re talking to patients about it you can make it seem much more true to how it actually is delivered.”
Current; Helping	56	*Building the identity of PR for patients* AB01H: “…we had already adopted this approach several years ago… ‘with regards to smoking it’s not an option, your lungs are telling you something, you’ve had a moment in time that your body’s not coping with the breathlessness so this is what you need to do to stop this happening’. And again with the rehab so we’ve introduced to say it’s an opt out… ‘so as part of your recovery we advise you that you need to come and do a pulmonary rehab programme because we know it’s gonna improve your breathlessness and if you don’t do it we know that the chances are you’re gonna become more breathless and more limited in what you can do each time you have a chest infection.’”	
Menu; Helping	24	*Building awareness of PR programmes* AB15H: “I think sometimes it’s quite nice, even if it’s a bit of written information that they have, just to ‘Have a little think about it cos there are various ways of doing this. And see if there’s anything there that you think would be of more use to you than another.’ And then they can ask the appropriate questions.”	
Current: Wish list	44	*Raising awareness of PR* AB11H: “I think we ought to call it something a bit more user friendly. Pulmonary rehabilitation, you think of drugs and alcohol. That’s what patients envisage as well and I think they don’t really understand what pulmonary means so I think that would really help. Calling it something different.”	
Menu; Wish list	36	*Development and dissemination of promotional materials for the menu of PR options* AB12H: “So perhaps having a cue card, so home-based and community-based pulmonary rehab, have one side—for a professional—have one side which explains what it is in plain language that you might use with a patient and then on the back have maybe like, you know, ‘It’s highly supported by evidence, the last Cochrane Review said this’. Like just one or two statement that really give you confidence.”	

Participants also identified ‘Insufficient knowledge of PR’ amongst HCPs and society in general. For HCPs, participants felt gaps in their knowledge regarding the complexity of PR (e.g., the eligibility criteria, programme logistics, menu of options, benefits) hindered patients’ understanding and ability to find meaning in PR because HCPs would be unable to translate their knowledge of PR effectively when introducing it to patients.

To combat these barriers, participants felt ‘Building the identity of PR for patients’ helped increase patient knowledge and understanding of PR beyond a brief verbal introduction. They described enablement strategies such as written and verbal information reinforced by pictures, disease education, media campaigns and standardised information online. This way PR, and COPD, became normalised within society and patient perceptions of COPD management. Participants also expressed the value of ‘HCPs’ knowledge, belief and experience of PR’. This was expressed as going beyond a basic awareness of PR and rather bringing them to life for the HCP. For example, HCPs felt observing a centre-based PR session had changed their perceptions of the programme and thereby enhanced their conversations with patients.

Furthermore, HCPs wanted to ‘Raise awareness of PR.’ They wished to redevelop the branding of PR to something understood by all (e.g., Breathe Easy was commended for its clear terminology), further increase its identity across society, and promote it as an active intervention for COPD management. HCPs also expressed a desire for PR education amongst HCPs (e.g., mandatory training for trainees).

Regarding the menu-based approach, not fully informing patients of the PR programmes was deemed unacceptable to patients as it could cause conflict in the HCP-patient encounter and reduce patients’ ability to make an informed decision about PR (‘Insufficient knowledge of the menu of programmes’). Contrastingly, participants recognised ‘Information overload’ as a barrier. When presented with numerous options or excess information, participants indicated poor health literacy could influence patients’ ability to make value-based decisions about PR.

To combat this, participants felt ‘Building awareness of PR programmes’ would help. Alike the current approach, participants felt written and verbal information reinforced by pictures and disease education would help to normalise the menu across society and thereby patient perceptions of COPD management. However, participants recognised this was not standardised or readily available. Therefore, they wished for the ‘Development and dissemination of PR promotional materials’ including visual, audio, written and online information to help educate patients, carers and HCPs about the menu of programmes. Complimentary to educational materials, participants recommended the development of decision-making tools to facilitate patients’ PR decision-making with HCPs.

### Perceived ability to access PR

For the current approach ‘A patient’s social circumstance’ captured the perceived barriers patients face when making decisions about PR (e.g., work commitments, caring roles, access to transport, vulnerability due to Covid-19; Table 4). To combat this and support patient access to PR, ‘Having PR programmes for all’ was recommended (i.e., a choice of home and centre-based PR). Additionally, participants recommended ‘Engaging in problem solving with patients’ to help identify and alleviate individuals’ barriers and thereby support patients to choose a programme which is both appealing and appropriate for their needs. For example, for patients without transport, HCPs recommended offering home-based programmes. Participants also felt ‘Having a targeted approach’ would increase patients’ acceptance of PR as HCPs could time their approach to when patients are motivated to improve their symptomology (e.g., newly diagnosed patients/recent inpatients). Furthermore, participants recognised the value of ‘Support from family and peers to access PR’. They felt family members reinforced the value of PR, increased motivation to attend and provided a safe environment for those completing home-based programmes. Peer support was beneficial for those unable to share health concerns or make health decisions with family.

**Table 4:** Perceived ability to access PR.

Frame of reference (current PR/menu-based approach pathway) and Item type	Participation rate (%)	Category	Sub-category
Current; Hindering	96	A patient’s receptiveness	*A patient’s social circumstances* AB12H: “COPD is a poor person’s disease and because of that you’ve got all the health economics and the kind of sociology all wrapped up in that… These patients probably have a lifetime of not accessing healthcare and this is just another thing for them not to access.”
Current; Hindering	84	Constraints of the PR service	*Constraints of PR delivery* AB14H: “I think that’s the biggest thing, the point at which you introduce it, the point at which they’re able to access it can be a barrier in itself. We can refer somebody, they can be offered a place that gap can change their motivation and the factors that will make them decide are they going to go or are they not.” *Constraints of PR referral process* AB17H: “Knowing who does the referral always used to be a problem. It’s difficult when you’re a trainee and you rotate round cos you get used to one way… in some hospitals I’ve worked in it has to be a primary care referral so you would write back to the GP and ask the GP to refer them. And then it would seem like either the GP hadn’t done it or the patient didn’t want to go but would say that they’d never been sent. So you would never really know cos you didn’t have access to any of the primary care records. And then when you work in a new centre you don’t really know who the person is, so you kind of write these letters like ‘Dear…’ to whoever ‘…Please [put name in] can you refer this patient for pulmonary rehab…’ so it’s probably just knowing who the person in your trust is…”
Current; Hindering	40	Constraints of the healthcare service	*The barrier of time* AB05P: “I think [HCPs’ are] so limited for time. You go in there and they haven’t got time to speak to you let alone anything else.”
Menu; Hindering	28	Constraints experienced by the healthcare service	*Time* AB04H: “I think if we are resourced well enough to spend half an hour for a COPD review then I think it’s possible… So whilst this is really positive, talking through all these options takes time and I think that’s your biggest factor here in what would stop a clinician in primary care from sharing all of this.”
Current; Helping	64	Having PR programmes for all	*PR design and delivery* AB02P: “…not everyone wants to come to hospital twice a week…they prefer to work from home. I’m the other way round, I had an office-based job and I enjoyed going to the office each day which is how I would view this rather than trying to get up in the morning as my son-in-law does and he works 100% from home on his job. Well I couldn’t do it, I’d find other things to do…” *PR accessibility* AB14H: “…our local rehab programme, that’s specifically pulmonary, happens at a local village hall. So, it’s in the adjacent village and I would say probably that most of our patients who attend pulmonary rehab, not exclusively but most of them, would choose to go to that venue because of the locality, because it’s geographically easier for them… familiarity of the people who might be there and for getting there.”
Current; Helping	32	Valued support	*Support from family and peers to access PR* Interviewer: “You said you received a letter?” AB22P: “Well I looked at the letter with my son and he said ‘Go for it Mam,’ so I filled it in and he posted it in the letterbox.”
Current; Helping	32	*Engaging in problem solving with patients* AB14H: “You get people that say they don’t like working in groups. But again you’ve got the alternatives, and that you can play to your advantage. Cos sometimes people say that thinking ‘well I’m out of jail…’ So they can say ‘Oh I don’t like working in groups,’ ‘Oh well if you don’t like working in a group, maybe you’d be interested in this programme you can do yourself at home. We’ll support you…’ So, sometimes people say it thinking it’s an out, but actually it’s an in. So yeah [we] can turn it around.”	
Current; Helping	32	*Having a targeted approach* AB17H: “If you’re trying to say, ‘Look you’ve got this symptom, it’s not responded to all of these other things you’ve tried. I really think that there’s good evidence that this is something that’s gonna get better with physio or with rehab’ then they’d be a bit more inclined to do it and they will usually try it.”	
Menu; Helping	28	*Engaging in problem solving* AB07H: “’If it’s not going to work with you coming into the classes here to do it, well can we get you closer to home? Can we get classes closer to home? Are you savvy enough to do it online?’ You know, rehab has evolved so much over the last 15 years… there’s lots of opportunities to involve patients some way in a rehab programme.”	
Menu; Helping	88	*Facilitating choice* AB06P: “You’ve made it available to me. I can’t drive, I’m working full time and it’s available.”	
Current; Wish list	72	Improving access to PR	*Improve how PR is delivered* AB19H: “I just want [there] to be an easier way to do it really. It would be good if there was something in-house either onsite or locally because people are very, very reluctant to go to the hospital or somewhere further.” *Improve the PR referral pathway* AB03H: “…it could even pop up, if the GP is thinking of referring to a Respiratory Consultant for advice with COPD, you know ‘Have you considered pulmonary rehab instead?’ Something like that and you can catch more patients.”
Menu; Wish list	56	Developing a PR service for all	*Continue to develop the service* AB25P: “Six months is a long time, do you know what I mean? It’s long enough but sometimes it might not be long enough for some people… It’s like, with myself, I’d just fool myself that I’m doing okay and I’m not… I don’t work cos of my disabilities and I can’t afford a personal trainer. Interviewer: Yeah. So you need that extra step… longer membership at the gym or another programme type thing? AB25: Yeah.”

Further barriers highlighted by participants included ‘Constraints of PR delivery,’ ‘The barrier of time,’ and ‘Constraints of the PR referral process.’ These recognised the organisational barriers to patients’ PR decision-making, for example, limited availability of programmes during Covid-19, inflexibility of programme delivery, inconsistent referral processes across sites and limited time in healthcare consultations for patients and HCPs to discuss PR.

To combat these barriers, participants wished to ‘Improve how PR is delivered’ by adapting programmes to suit disadvantaged populations (e.g., those with work commitments, caring roles, etc.), introducing local venues for centre-based PR, providing reliable transport services for centre-based PR, and facilitating greater social support for patients considering home and centre-based PR. They also wished to ‘Improve the PR referral pathway.’ They suggested HCP interventions (e.g., standardising electronic referrals, using computer pop-ups to initiate PR discussions with patients) and patient interventions to build self-efficacy (e.g. self-referrals, negotiating a start date at referral).

For the menu-based approach participants did not identify any barriers related to patients’ perceived ability to access PR via the menu-based approach. Instead, they felt by offering a menu of home-based and centre-based evidence PR programmes would increase patient appeal and enable greater perceived access (‘Facilitating choice’). However, participants felt ‘Continuing to develop the service’ was important to continue to support patients’ PR decision-making and uptake to the service. They suggested developing new programmes to suit individual patient groups, creating opportunities for programme switching and the development of maintenance programmes/extensions for PR graduates.

### A desire to accept PR

For the current approach ‘A patient’s health and their experience of healthcare’ were identified barriers to PR decision-making, and thereby acceptance of PR (Table 5). Participants recognised patients’ health experiences positively and negatively influence many patient decisions. For example, they felt poor health status would reduce patients’ acceptance of PR when it is perceived as incongruent to their recovery.

**Table 5:** A desire to accept PR.

Frame of reference (current PR/menu-based approach pathway) and Item type	Participation rate (%)	Category	Sub-category
Current; Hindering	96	A patient’s receptiveness	*A patient’s beliefs* AB07H: “Patients, particularly with COPD, are really hard on themselves. ‘I’ve smoked, I deserve it’ or ‘I smoked, I didn’t know…’ they almost apologise sometimes for ‘Well I tried rehab before. I don’t want to waste anybody more resources.’” *A patient’s health and their experience of healthcare* AB01H: “…when you’re feeling unwell in hospital the last thing you want to think about [is PR] and I think that’s always been our barrier that we’re approaching people that are feeling breathless and terrible and they don’t want to think about anything.”
Current; Helping	56	*Making PR meaningful to patients* AB01H: “I’ve told that story lots of times about the patient that couldn’t walk for 2 min and [after PR] could walk for 15 min. And you can see sort of a lightbulb… people thinking ‘Well you know what I can only walk for 2 min.”	
Current; Helping	60	*Patient receptiveness to PR referrals* AB09P: “I’ve got a brother in law who’s about two years older than me and he’s had COPD, well emphysema same as me for about four years, and he’s never attempted to do anything about it. He’s just sort of gone down and down and down with it. I mean now he’s on oxygen fifteen hours a day and I don’t wanna get like that.”	

Similarly, ‘A patient’s beliefs’ were highlighted as a hindering item. This referred to illness beliefs likely developed from health experience and which influence patients’ internal motivation for COPD self-management. Participants felt PR decision-making and thereby engagement in PR were challenged by patients’ negative perceptions of breathlessness, COPD, and low self-efficacy.

In contrast, ‘Patient receptiveness to PR referrals’ was considered beneficial to PR decision-making. Participants felt individual patient characteristics predicted PR acceptance and positive COPD self-management, (e.g., fear of ill health, high self-efficacy, prior positive experience of PR/exercise). However, participants considered these characteristics to be inconstant and that many factors could influence them.

Additionally, participants identified ‘Making PR meaningful to patients’ as valuable for patients’ PR decision-making. Participants believed this was achieved through patient-centred discussions to identify individual healthcare goals before introducing PR and discussing how or if PR could meet these goals. HCPs recommended using patient stories to “break the ice” and build patients’ confidence in accepting PR.

### Supporting the offer

For the current approach participants highlighted ‘Varying healthcare priorities’ within COPD management, particularly regarding treatment focus, being a barrier to HCPs offer of PR. Participants felt the prioritisation of PR varied across disciplines, which sent conflicting messages to patients regarding its value (Table 6).

**Table 6:** Supporting the offer.

Frame of reference (current PR/menu-based approach pathway) and Item type	Participation rate (%)	Category	Sub-category
Current; Hindering	40	Constraints of the healthcare service	*Varying healthcare priorities* AB05P: “Well my daughter was given lots of papers to talk about COPD. She was given 4 monthly check ups… She was sent to xxx for her breathing exercises…the only thing I had was two steroids and a yearly visit from the nurse.”
Current; Hindering	36	*A lack of HCP–patient relationship* AB17H: “…if I’m seeing somebody on a ward I don’t know that well but just fits into a particular category… a patient with COPD who’s come in with an infectious exacerbation then you say ‘There’s good evidence for pulmonary rehab so at some point, maybe not now but when you’ve recovered, it would be a good idea for you to do this…’ Whereas, with some of the asthma patients… you know a particular element of their symptoms is due to deconditioning and their breathlessness isn’t responding to things like inhaled steroids then you might spend a bit more time going through it and saying ‘This is why I think you particularly might benefit from this cos it will build your muscle strength and over time I think you’ll see an improvement in that particular symptom.’”	
Menu; Hindering	36	*The beliefs of HCPs* AB13H: “I personally think physically going to a group run by physios with other people there and turning up on the day I would be more likely to maintain the exercise…”	
Current; Helping	48	HCP engagement in PR	*Facilitating a joined-up service* AB12H: “Because of the type of intervention it is they need to be empowered to do it… so sometimes trickling [the idea of PR] in and definitely putting on my plan to revisit and revisit and revisit… a really good example would be that somebody else has gone along, normally a COPD nurse specialist, and they’ve spent loads of time talking about inhaler technique and maybe future care planning but they haven’t spent that much time around pulmonary rehab and I can go along sometimes and because of the slight different perspective, slight different training, can change their view on that and get them in a different way.”
Current; Helping	32	Valued support	*Support from HCPs* AB07H: “…you’re sort of left on your own [when completing home-based PR] and [patients] could do with a bit of interaction. So it’s just making them aware that as part of that, ‘Oh that’s fine but you can call the rehab office and they’ll help you if you’re not sure about anything or if you’re worried about something.’”
Current; Helping	44	*The HCP–patient relationship* AB04H: “I think the person who’s actually counselling them to go on pulmonary rehab. What sort of relationship do they have with that particular professional? What’s their consultation skills like? You know, cos consultation skills and being in synch with somebody’s health behaviours and psychology I think is really, really important… So if you’re gonna have somebody completely out of the blue trying to counsel patients to go to pulmonary rehab it won’t work as well as perhaps as having somebody who knows the patient, who has a trust with the patient and they’ve got a rapport built in.”	
Current; Wish list	72	Improving access to PR	*Improve how PR is offered* AB08P: “I’ve learned I can perhaps do something about [my COPD]… I can do that and try and help myself or other people can help me as well… If I’d seen this about 8 years ago [I] might have… I’ve never heard of it.”
Menu; Wish list	56	Developing a PR service for all	*Introduce the menu of programmes to all* AB15H: “I think it’s nice before they actually come to have some awareness…to say that ‘This is something that’ll be discussed when you go for your assessment, but these are the different options that are available. So have a think about what you think might work for you.’”
Menu; Wish list	48	*Guiding patient decisions* AB24P: “…it’s okay giving you all these choices but you do need somebody to go through it with you…”	

Similarly, participants reflected on the sheer quantity of content to cover with patients during COPD consultations. They felt this could alter a consultation’s focus and result in a check-box exercise instead of a patient-centred discussion, in consequence creating **‘**A lack of HCP–patient relationship.’ This relationship was described as fragile, and easily dashed when patients felt alienated from their HCP. Participants therefore recognised the value of a positive ‘HCP–patient relationship’ built upon continuity of care and trust to facilitate informed and value-based PR decision-making.

Participants also described the value of ‘Support from HCPs’ which involved highlighting opportunities for continued support from HCPs and other HCPs to reassure and encourage patient engagement in PR. Furthermore, ‘Facilitating a joined-up service’ was praised for creating a supportive environment for PR uptake and normalising PR discussions across disciplines. Participants felt “trickling the idea in” by introducing PR from a multi-disciplinary team helped to normalise it within patient expectations of COPD management. However, participants felt more effort was needed to ‘Improve how PR is offered’ by engaging multi-disciplinary teams in PR discussions with patients in a timelier way.

For the menu-based approach, participants felt supporting the offer of PR was challenged by ‘The beliefs of HCPs.’ HCPs felt a preference for centre-based programmes because of its familiarity, the face to face supervision it provides and anticipated patient non-compliance for the home-based PR programmes. This therefore introduced bias when discussing the menu of programmes with patients. A solution proposed was ‘Guiding patient decisions.’ Participants wished for HCPs to introduce suitable PR programmes to patients to facilitate patient-centred PR decision-making. They acknowledged the level of guidance patients need may vary, with some happy to take an active role in sharing the decision and others preferring greater input from HCPs.

Moreover, participants wished to integrate the menu of options into standard practice (‘Introduce the menu of programmes to all’). However, there was disagreement about when it should be introduced (e.g., at referral or at PR assessment), which was dependent on time available for meaningful patient PR discussions.

## Discussion

An appraisal of a current PR approach and a newly proposed menu-based approach pinpointed five high level themes representing the influencers of patient decision-making and thereby acceptance of PR; Understanding PR, perceived ability to access PR, a desire to accept PR, supporting the offer, and understanding COPD.

Insufficient knowledge of PR and COPD across patients, HCPs and society appeared to compromise PR decision-making within healthcare consultations. For patients, the complexity of COPD proved a barrier to positive self-management as participants described patients steep learning curve extending well beyond diagnosis. Similarly, whilst most patients are aware of COPD, studies have shown over half of patients feel they need more information^21,22^. The way information is framed to patients also proves problematic, particularly the use of medical COPD jargon and stigmatising PR terminology^23,24^. Our findings highlight a need to increase patients’ health literacy to facilitate positive COPD decision-making (i.e., engaging in PR). Participants recommended the development of standardised PR promotional materials to raise awareness of PR, a need observed by other PR services^24,25^ with evidenced success in primary care^26^.

Insufficient knowledge hindered the ability of HCPs to translate PR and support decision-making with their patients. Consonant with the literature, insufficient knowledge has been associated with expressing PR with conviction^27,28^, perhaps influenced by misconceptions of COPD management, including a lack of belief in exercise therapy^25^, poor adherence to COPD guidelines and a lack of COPD-specific training^29^. In primary care, this has been attributed to most COPD management being delegated to Practice Nurses, leaving them feeling isolated and GPs feeling de-skilled^30^. Participants advocated for HCPs to develop a more comprehensive understanding of PR, achieved through physical exposure to a programme. Alternatively, the provision of training on the benefits of exercise therapy has been proposed to promote the role of exercise in COPD management^25^.

Participants described an accumulation of social factors which hinder patients perceived access and acceptance of PR. Work and family commitments have been shown to limit patients’ access to PR^7,27,31^, as it is perceived to be time-consuming and in conflict with established routines^32^, suggesting PR may not be valued by patients. Negative illness beliefs including low self-worth, fear and poor motivation all appear to reduce PR acceptance and compliance^7,27,31–33^. Similarly, we observed poor health status as a barrier, however, it is possible this limits patient recall of PR discussions rather than acceptance^22,32^.

Participants promoted a choice of centre and home-based PR programmes to meet individual patient needs, facilitate decision-making and provide greater access. They wished to standardise the menu within PR discussions, a unique finding, however, a consideration for services who also suffer poor uptake to centre-based programmes. Furthermore, engaging in patient-centred discussions to make PR meaningful to patients was recommended. Indeed, introducing PR as an intervention enabling patients to do meaningful activities has shown to increase motivation and acceptability of PR^30^.

Constraints of the referral process, limited availability and inflexibility of programmes were all reported barriers. One example to combat this was streamlining referrals (e.g., using an electronic pathway). Interventions to improve the availability and flexibility of PR delivery are lacking, however, there is some evidence of interventions to improve patient referrals. A systematic review including 10 studies found four interventions which reported statistically significant increases in PR referrals^34^; a patient-held quality scorecard^35^, a primary care education programme^36^, a collaborative model of education and change implementation^37^, and mandatory monitoring of quality indicators in outpatient departments^38^.

A disconnect in the HCP–patient relationship, was identified as creating conflict during consultation. Communication barriers and paternalistic consultation styles have shown to stop HCPs from offering PR^30,39^. Likewise, in an era of increasing healthcare pressure, PR conversation quality is reduced^24,30^. Participants recognised time may always be a barrier but recommended using it effectively in patient consults to develop therapeutic relationships, build understanding, self-efficacy and motivation and support PR decision-making (i.e., with patient stories, motivational interviewing).

Whilst this approach was appraised favourably, HCPs beliefs about the equality of programmes and concerns about patient motivation during unsupervised programmes was identified. Similarly, referrers attitudes to PR has shown to attribute positive or negative value to it^30^ which can create barriers to acceptance^32^. To alleviate bias, participants recommended increasing HCPs understanding and belief in the menu of options using decision support tools to structure PR conversations and continuing efforts to develop programmes which meet the complex needs of patients.

Health literacy was also felt to negatively influence patients’ ability to absorb the menu, understand it and make a definitive programme choice. The amount of information presented to patients was a specific concern. Participants recommended the development of standardised mid-low literacy materials to advertise the menu and a decision-making tool to be used with HCPs to guide patients’ PR decision-making.

These findings demonstrate an appetite for supporting patients’ PR decision-making, particularly via tools which facilitate knowledge translation and guide the decision-making process. One approach is patient decision aids as these present evidence-based information in such a way to improve patients’ health literacy, whilst minimising cognitive load or bias, clarify the menu of options and highlight the associated risks and benefits of each, and prompt patients to attribute personal meaning to each option^40^. Patient decision aids are evidenced across a variety of healthcare settings for increasing patients’ knowledge and perceptions of risk and reducing any feelings of internal conflict and passivity^41^. They are therefore considered valuable tools to enable patients, with support from their HCP and family, to reach value-based and informed decisions^42^. There are currently no patient decision aids to support patients’ PR decision-making.

These results offer valuable insight into patients’ decision-making needs for PR and provide guidance for the continued evolution of services to meet the populations’ needs. Additionally, this is the first PR study using the Enhanced Critical Incident Technique methodology, extrapolating specific and comprehensive barriers, facilitators, and improvements to patients’ PR decision-making.

Importantly, our interviewees were all White British and were from a single centre offering specific PR programmes. We acknowledge that our findings may not reflect the barriers and facilitators experienced by individuals from different cultural, organisational, or healthcare contexts.

Our decision to include only participants who were naïve to PR allowed for greater reflection on patients’ decision-making processes, however, it may have omitted the role of PR experience in patients’ decision-making. Additionally, we asked participants to reflect upon a real and hypothetical PR conversation and then compared the results of both. Without direct experience of a menu-based approach introduction, it is possible patient and HCP interpretations of this differed.

Minor amends to the Enhanced Critical Incident Technique credibility checks were made, including, interview fidelity was assessed by reviewing transcripts, one category’s participation rate was less than the proposed threshold and only 36% of participants cross-checked their results (Supplementary material). Furthermore, whilst it was intended to collect equal amounts of critical incidents for the current and the menu-based approach, more incidents were obtained from the current PR approach. We believe this is because of its greater familiarity to our participants. Future exploration of the decision-making needs of patients using the menu-based approach will be needed to inform the feasibility of supporting interventions.

Consonant with Early and colleagues recommendations^34^, we recommend future research focus upon developing, evaluating and reporting high-quality interventions to increase referral uptake. In addition, we recommend programme adaptations to increase patient accessibility and acceptability of PR and shared decision-making interventions (e.g., a patient decision aid, decision coaching) to facilitate patient-centred, meaningful PR decision-making.

A menu-based approach to PR increases the opportunity for patients to engage. However, insufficient knowledge and understanding of the menu limits its perceived feasibility and acceptability. Knowledge translation and decision guidance tools such as a patient decision aid has the potential to increase patient and HCPs knowledge of the menu of PR programmes, elicit patient-centred discussions and facilitate informed and value-based PR decision-making.

## Supplementary information

Supplementary material

REPORTING SUMMARY

## Data Availability

The datasets generated during and/or analysed during the current study are available from the corresponding author on reasonable request.
