# Supplementary material for: A qualitative assessment of the pulmonary rehabilitation decision-making needs of patients living with COPD
Source: NPJ Prim Care Respir Med. 2022 Jun 29;32:23. doi: 10.1038/s41533-022-00285-9 (PMC9243001; doi:10.1038/s41533-022-00285-9)
Supplement: Supplementary file 1 — Supplementary material [file 41533_2022_285_MOESM1_ESM.pdf]

Supplementary Materials

Supplementary material 1: Interview Guide – Healthcare Professionals

Participant #: ______________________

Date: _____________________________

Interview Start Time: ________________

1. Contextual Component

Preamble: As you know, I am investigating how the Pulmonary Rehabilitation, or PR, pathway is experienced by those who refer patients to the service. The purpose of this interview is to collect information about your experiences of talking to patients about PR, any challenges you may have faced with this and your ways of dealing with it. Thank you for agreeing to talk with me today. Are you happy for me to audio-record our discussion? Your comments will remain anonymous and confidential.

a. As a way of getting started, perhaps you could tell me a little bit about your local PR programme/(s)?

b. You volunteered to participate in this study because you identified yourself as a person who refers patients to PR. Can you tell me how you introduce patients to the PR programme?

c. In your experience, how do patients respond to your introduction of PR?

d. Over time, have you refined the way you speak to patients about PR based upon their responses? If yes: In what way?

2. Critical Incident Component Transition to Critical Incident questions:

You said that patients have a … response with regards to the introduction of the PR programme.

a. What do you think has helped to engage patients with PR? (Probes: What created the biggest impact? How has it created that impact? Can you give me a specific example of this?)

b. Are there things that have made it more difficult for you to engage patients in PR? (Alternative question: In you experience, are there any obstacles you have faced in engaging patients with PR?) How did 'obstacle' hinder? Tell me what it was about 'obstacle' that you find so unhelpful.

c. We've talked about what's helped to engage patients in PR (name them), and some things that have made it more difficult for you to engage patients (name them). Are there other things that you think would help you to continue patient engagement with PR? (Alternative question: I wonder what else might be helpful for you that you haven't had access to? How would it help? Tell me what it is about … that you would find so helpful.) Example (In what circumstances might this be helpful?)

PAUSE: Show menu-based approach prompt

a. How do you think this would help to engage patients with PR? (Probes: What would create the biggest impact? How would it create this impact? Can you give me a specific example of this?)

b. Are there things that would make it more difficult for you to engage patients in PR? How would 'obstacle' hinder? Tell me what it is about 'obstacle' that you would find so unhelpful.

c. At what time point do you think these options should be introduced to a patient? (Prompt: referral or assessment, by whom?).

d. How do you imagine a conversation about the menu of PR options might go with a patient? (Prompt: Do you foresee any positives/negatives? If so what are they? How might the conversation go at the moment because of Covid-19?)

3. Demographics Component

1. Occupation:____

2. Number of years in this occupation:____________________________

3. Length of time in current job:________________________________

4. Number of years in this industry:______________________________

5. Age:____

6. Gender:____

Interview End Time: ___________________________

Length of interview: _____________________

Interviewer's Name: _______________________

Supplementary material 2: Interview Guide – Patients

Participant #: _____________________

Date: ___________________________

Interview Start Time: _______________

1. Contextual Component

Preamble: As you know, I am looking into how 'Pulmonary Rehabilitation, or PR, is understood and experienced by patients with COPD. The purpose of this interview is to collect information about your understanding of PR and what you think about a new 'menu-based approach' to it. Thank you for agreeing to talk with me today. Are you happy for me to audio-record our discussion? Your comments will remain anonymous and confidential.

a. As a way of getting started, perhaps you could tell me a little bit about how you were first introduced to PR?

b. From this introduction, can you tell me what you think PR is likely to involve?

PAUSE: (show menu-based approach prompt).

c. What are your thoughts on the different types of PR you could take part in? Follow up questions: Were these options offered to you when you were first introduced to PR?

d. If yes: Did you like to see or hear about these options of PR when you were introduced to them? If no: Would you have liked to see or hear about these options of PR when you were introduced to them?

e. Would you like to hear more about these PR options at your assessment appointment with a PR specialist?

2. Critical Incident Component Transition to Critical Incident questions:

You said that you would/would not have liked to see/hear about all the options of PR you could take part in.

a. How do you think having more choices of PR might help you? (Probes: What would be the biggest help for you? Can you give me a specific example of this?)

b. Are there things that would make it difficult for you to decide which PR you want to do? (Alternative question: Are there any disadvantages to having these different choices about your PR?) How would this 'disadvantage' hinder? Tell me what it might be about this 'disadvantage' that you would find so unhelpful.

c. We've talked about the pros and cons of having more choice about PR (name them). Are there other things that you think could help you? (Alternative question: I wonder what else might be helpful for you that you haven't had access to? How would it help? Tell me what it is about 'thing' that you would find so helpful. In what circumstances might this be helpful?)

3. Additional contextual component (if not already discussed)

As we are living through a global pandemic it would be helpful to hear what you would like from you PR team right now.

a. Could you tell me what you expect or what you would like from the PR team at the moment?

b. What are your thoughts on the available options now, baring in mind centre and community-based PR has been suspended due to social distancing measures? (Prompt: How would you feel about doing home-based PR right now?

How would you feel about remote care from the PR team? How would you feel about exercising at home right now? Are there any positives/negatives of doing home-based PR for you?)

3. Demographics Component

1. Age:____

2. Gender:____

3. Age when diagnosed with COPD:______________________

4. Previous attendance at Pulmonary Rehabilitation programme:__Y/N__

5. Offered menu-based approach at introduction to PR:________Y/N____

Interview End Time: ______________________

Length of interview: ______________________

Interviewer's Name: ______________________

Supplementary material 3: Menu-based approach prompt

Hospital-based and community-based Pulmonary Rehabilitation

What is Pulmonary Rehabilitation?

Pulmonary Rehabilitation is a programme of exercise and education for patients with a chronic respiratory disease, including COPD.

How is it delivered?

The programme is run twice a week for 6 weeks at the following locations: Glenfield Hospital, Leicester General Hospital, the National Centre for Sport and Exercise Medicine at Loughborough University and other community venues across Leicestershire. It is run by a friendly team of healthcare professionals, known as the Pulmonary Rehabilitation team. These include nurses, physiotherapists, and occupational therapists.

What happens in each session?

The Pulmonary Rehabilitation team will show patients how to increase their functional exercise capacity safely and effectively. The first half of the session involves carrying out physical exercise. The exercise is carefully planned to suit each patient's abilities so that it provides just the right amount of activity for them. The other half of the session is spent discussing information and advice about living with their respiratory disease and showing patients how to get the most out of their life.

Pulmonary rehabilitation helps to:

- Reduce patients feelings of breathlessness
- Improve their general health and level of fitness
- Improve their ability to carry out the daily activities that are important to them
- Understand their respiratory disease
- Control the symptoms of their disease
- Improve their levels of confidence and quality of life

This programme works well for patients who:

- Want direct face to face and regular support from healthcare professionals
- Enjoy being in a group setting
- Want to meet other people are living with a respiratory disease

SPACE for COPD

What is SPACE for COPD?

SPACE for COPD is a manual-based self-management programme of exercise and education for patients with COPD.

How is it delivered?

Every patient is assessed by the Pulmonary Rehabilitation team on an individual basis to calculate their current abilities and needs, both physically and mentally. The SPACE for COPD manual is introduced to patients by a respiratory healthcare professional.

At home, the patient works through the manual independently with telephone support from their respiratory healthcare professional. They are encouraged to set goals and progress through a prescribed training programme to achieve their weekly targets. Exercises include a daily walking regime and resistance training. The patient works through the manual at their own pace.

SPACE for COPD helps to:

- Reduce patients feelings of breathlessness
- Improve their general health and level of fitness
- Improve their ability to carry out the daily activities that are important to them
- Understand their respiratory disease
- Control the symptoms of their disease
- Improve their levels of confidence and quality of life

This programme works well for patients who:

- Would like to learn how to better manage their disease with telephone support from healthcare professionals
- Have other important commitments e.g. they are a carer, work
- Live far away from the PR venues and have difficulty getting to them

Web-based SPACE for COPD

What is web-based SPACE for COPD?

Web-based SPACE for COPD is an online programme of exercise and education for patients with COPD

How is it delivered?

Every patient is assessed by the Pulmonary Rehabilitation team on an individual basis to calculate their abilities and needs, both physically and mentally. The web-based SPACE for COPD programme is introduced to the patient by a respiratory healthcare professional. A username and password is given to the patient so they can access the programme from a computer or tablet at their home.

At home, the patient works through the programme independently online. They receive email prompts and telephone support from their respiratory healthcare professional. The patient is encouraged to set goals and progress through a prescribed training programme to achieve their weekly targets. Exercises include a daily walking regime and resistance training. The patient works through the web-based programme at their own pace.

Web-based SPACE for COPD helps to:

- Reduce patients feelings of breathlessness
- Improve their general health and level of fitness
- Improve their ability to carry out the daily activities that are important to them
- Understand their respiratory disease
- Control the symptoms of their disease
- Improve their levels of confidence and quality of life

This programme works well for patients who:

- Are confident in using computers
- Live far away from the PR venues and have difficulty getting to them
- Have other important commitments e.g. they are a carer, work
- Would like to learn how to better manage their disease with telephone and email support from healthcare professionals

Active Lifestyles scheme

What is the Active Lifestyles scheme?

The Active Lifestyle Scheme supports inactive people with a medical condition to build regular and sustainable exercise into their everyday lives.

How is it delivered?

The Active Lifestyle Scheme is delivered across council-run leisure centres in Leicestershire. Individuals can use the most convenient centre to them at any one time.

Under the supervision of qualified instructors, each individual will be offered the guidance and support they require to enable them to take the first steps to leading an active and healthy lifestyle.

Active Lifestyles helps to:

- Manage weight
- Improve muscle and joint functions
- Improve overall mood
- Manage and control existing conditions
- Protect patients from developing other health complications too.

This programme works well for patients who:

- Have previously completed PR and would like support to continue to exercise after their PR classes

Breathe Easy

What is Breathe Easy?
Breathe Easy groups provide support and information for people living with a lung condition, and for those who look after them.

How are they delivered?
There are more than 230 Breathe Easy groups throughout the UK. The groups range from support groups, to exercise classes and singing classes.

The Breathe Easy groups are run by their members who meet usually once every month.

Breathe Easy groups helps individuals to:
- Increase their exercise abilities
- Make new friends who know what they're going through
- Learn more about living with a lung condition.

This programme works well for patients who:
- Would like continued group support to exercise and stay healthy
- Would like to be part of a community of patients who also have respiratory diseases

Supplementary material 4: Credibility check adherence

Credibility checks	Recommended actions	Our actions
Audiotaping interviews	To audiotape the data collection interviews.	We audiotaped and transcribed verbatim all participant interviews.
Interview fidelity	To have an expert in the ECIT research method listen to every third or fourth taped interview. Feedback is then provided to the interviewer prior to conducting the next interview.	The first two participant transcripts were reviewed by author ML who has expertise in ECIT analysis and feedback provided.
Independent extraction of critical incidents	To have an individual other than the person who initially identified CIs to review and independently identify (or extract) what they think are the CIs. Butterfield et al.[39] suggest it is customary to randomly choose 25% of the transcripts to give to this independent individual. Once the person has extracted what he or she thinks are the CIs, the researcher compares what each has extracted and computes the percentage of agreement. For incidents that do not match, the researcher and independent reader can discuss the discrepancy for the purpose of resolving the difference. If no resolution can be made, the concordance rate would reflect less than a 100% match, and the incident in question would not be used in further analysis.	Author BA independently coded 25% of transcripts to identify critical incidents. Discrepancy in coding was discussed and minor amends were made to the wording of 8 critical incidents. No critical incidents were removed, and an additional 15 were added.
Keeping track of category exhaustiveness	To keep a log of each interview as its CIs are placed into the category scheme being created.	We maintained a record of category scheme formation throughout analysis to capture the time point of category scheme exhaustion.
Participation rates	To count the number of different participant numbers under each category and divide that number by the total number of participants to determine the participation rate. A minimum of 25% participation is recommended by Borgen and Amundson[40].	We calculated participation rates and presented these for each category (see results). All categories were retained (including one category with 24% participation rate) as we observed it across appraisals of both the current and menu-based approach pathway.
Placing incidents into categories by an independent judge	To have an independent person place 25% of the CIs into the categories that have been created by the researcher and to calculate the match rate between that person's placements and those of the researcher.	Author CB, independent to data collection and analysis, was sent 25% of the critical incidents within each category, the category headings and the operational definitions and asked to categories them. A

	A match rate of 80% is recommended[41].	discrepancy rate of 10% was considered acceptable.
Cross-checking by participants	To invite participants to review the interpretations made by the researcher to ensure they are supported by study participants, thus confirming the CIs extracted from the transcripts. The participant is asked to review the list of CIs and then answer the following questions:<br>1. Are the helping/hindering CIs correct?<br>2. Is anything missing?<br>3. Is there anything that needs revising?<br>4. Do you have any other comments?<br>The participant is then asked to review the categories into which the CIs have been placed and to answer the following questions:<br>1. Do the category headings make sense to you?<br>2. Do the category headings capture your experience and the meaning that the incident or factor had for you?<br>3. Are there any incidents in the categories that do not appear to fit from your perspective? If so, where do you think they belong?<br>Additionally, to ask follow up questions that arose from the participant's first interview. This is most often needed when something in the first interview sounded like it was a CI, but the supporting importance or example information was missing. Assuming the supporting details are provided, the item is then coded, added to the total number CIs, and added to the appropriate category. The researcher would advise the participant of the category into which the incident will be placed to obtain the participant's agreement.	We gave participants 6 weeks to review the critical incidents extracted from their transcripts and review their placements within the generated categories. The following questions were asked of participants:<br>• Are the items correct?<br>• Is anything missing?<br>• Is there anything that needs changing?<br>• Do you have any further comments?<br>• Do the category headings make sense to you?<br>• Do the category headings capture your experience and the meaning the critical incidents and wish list items had for you?<br>• Are there any critical incidents in the categories that do not appear to fit your views? If so, where do they belong?<br>Responses were received from 36% of participants (1 participant had died meaning only 24 participants were contacted). One participant asked for the wording of a critical incident to be amended slightly. Otherwise, participants agreed with the proposed analysis. No follow up questions were required.
Expert opinions	To submit the categories that have been created to two or more experts in the field. The experts are asked to provide responses to three broad questions after reviewing the categories:<br>1. Do you find the categories to be useful?<br>2. Are you surprised by any of the categories?<br>3. Do you think there is anything missing based on your	We submitted the category scheme to author ML (an expert in the field of ECIT analysis) and author SS (an expert in the field of PR). The categories were deemed congruent with their knowledge and minor amends were made to rename one sub-category and remove sub-categories from four main categories as it was felt these did not need to be split any further.

	experience?	
Theoretical agreement	To review scholarly literature in appropriate disciplines to ensure the data generated aligns with two theoretical agreements:<br>1. Assumptions underlying the study<br>2. Emergent category relationships with the literature (e.g., congruency, incongruency).	Prior to data collection, and based upon a systematic review of the literature, we developed the following hypotheses:<br>• There are barriers to PR access and patient decision-making.<br>• A menu-based approach to PR may facilitate better patient decision-making and engagement in PR.<br>Following data collection, we compared the generated categories to current PR literature. The congruency and incongruency of generated categories are reviewed in the discussion.
