# Supplementary material for: A qualitative assessment of the pulmonary rehabilitation decision-making needs of patients living with COPD
Source: NPJ Prim Care Respir Med. 2022 Jun 29;32:23. doi: 10.1038/s41533-022-00285-9 (PMC9243001; doi:10.1038/s41533-022-00285-9)
Supplement: Supplementary file 2 — REPORTING SUMMARY [file 41533_2022_285_MOESM2_ESM.pdf]

nature portfolio

Corresponding author(s): Amy Barradell

Last updated by author(s): May 6, 2022

Reporting Summary

Nature Portfolio wishes to improve the reproducibility of the work that we publish. This form provides structure for consistency and transparency in reporting. For further information on Nature Portfolio policies, see our Editorial Policies and the Editorial Policy Checklist.

Statistics

For all statistical analyses, confirm that the following items are present in the figure legend, table legend, main text, or Methods section.

n/a	Confirmed	
☒	☐	The exact sample size (n) for each experimental group/condition, given as a discrete number and unit of measurement
☒	☐	A statement on whether measurements were taken from distinct samples or whether the same sample was measured repeatedly
☒	☐	The statistical test(s) used AND whether they are one- or two-sided<br>*Only common tests should be described solely by name; describe more complex techniques in the Methods section.*
☒	☐	A description of all covariates tested
☒	☐	A description of any assumptions or corrections, such as tests of normality and adjustment for multiple comparisons
☒	☐	A full description of the statistical parameters including central tendency (e.g. means) or other basic estimates (e.g. regression coefficient) AND variation (e.g. standard deviation) or associated estimates of uncertainty (e.g. confidence intervals)
☒	☐	For null hypothesis testing, the test statistic (e.g. F, t, r) with confidence intervals, effect sizes, degrees of freedom and P value noted<br>*Give P values as exact values whenever suitable.*
☒	☐	For Bayesian analysis, information on the choice of priors and Markov chain Monte Carlo settings
☒	☐	For hierarchical and complex designs, identification of the appropriate level for tests and full reporting of outcomes
☒	☐	Estimates of effect sizes (e.g. Cohen's d, Pearson's r), indicating how they were calculated

Our web collection on statistics for biologists contains articles on many of the points above.

Software and code

Policy information about availability of computer code

Data collection	No software used
Data analysis	No software used

For manuscripts utilizing custom algorithms or software that are central to the research but not yet described in published literature, software must be made available to editors and reviewers. We strongly encourage code deposition in a community repository (e.g. GitHub). See the Nature Portfolio guidelines for submitting code & software for further information.

Data

Policy information about availability of data

All manuscripts must include a data availability statement. This statement should provide the following information, where applicable:

- Accession codes, unique identifiers, or web links for publicly available datasets
- A description of any restrictions on data availability
- For clinical datasets or third party data, please ensure that the statement adheres to our policy

The datasets generated during and/or analysed during the current study are available from the corresponding author on reasonable request.

Field-specific reporting

Please select the one below that is the best fit for your research. If you are not sure, read the appropriate sections before making your selection.

☐ Life sciences ☒ Behavioural & social sciences ☐ Ecological, evolutionary & environmental sciences

For a reference copy of the document with all sections, see nature.com/documents/nr-reporting-summary-flat.pdf

Behavioural & social sciences study design

All studies must disclose on these points even when the disclosure is negative.

Study description	Qualitative research methods.
Research sample	Patients were eligible if they had a confirmed diagnosis of COPD (post bronchodilator FEV1/FVC ratio <70%), had received a PR referral and had not previously undertaken PR. This allowed reflection upon the decision-making processes for PR rather than prior experience of a PR programme. HCPs were eligible if they had been actively referring patients to the PR service for a minimum of 1 year. This ensured adequate experience to reflect upon.
Sampling strategy	We used the proportionate allocation method of stratified sampling to recruit participants representative of our service. For patients, our sampling considered referral setting (e.g. inpatient, outpatient, GP setting) and residence (e.g. inner-city, urban). For HCPs, our sampling considered referral setting (e.g. primary care, secondary care) and site location (e.g. inner-city, urban).
Data collection	Following participant consent, the first author, a Health Psychology PhD student, collected baseline contextual data and conducted semi-structured interviews face to face or via telephone. Following the Covid-19 pandemic, we added additional contextual questions to the interview guides (Supplementary material) and continued beyond the proposed sample size. Each interview was digitally recorded and transcribed verbatim.
Timing	Data collection began in July 2019 and ended in October 2020.
Data exclusions	No data excluded from analysis.
Non-participation	85 patients were invited to be interviewed (response rate = 12.9%)<br>34 healthcare professionals were invited to be interviewed (response rate = 41.1%)
Randomization	Randomisation not appropriate.

Reporting for specific materials, systems and methods

We require information from authors about some types of materials, experimental systems and methods used in many studies. Here, indicate whether each material, system or method listed is relevant to your study. If you are not sure if a list item applies to your research, read the appropriate section before selecting a response.

Materials & experimental systems

n/a	Involved in the study	
☒	☐	Antibodies
☒	☐	Eukaryotic cell lines
☒	☐	Palaeontology and archaeology
☒	☐	Animals and other organisms
☐	☒	Human research participants
☐	☒	Clinical data
☒	☐	Dual use research of concern

Methods

n/a	Involved in the study	
☒	☐	ChIP-seq
☒	☐	Flow cytometry
☒	☐	MRI-based neuroimaging

Human research participants

Policy information about studies involving human research participants

Population characteristics	See above.
Recruitment	All participants were contacted by an invitation letter or email. Those who expressed interest were contacted to arrange a convenient date and time for interview.
Ethics oversight	We received ethical approval by East Midlands – Leicester South Research Ethics Committee (REC: 17/EM/0156), the Health Research Authority and the research site. Participants provided written informed consent.

Note that full information on the approval of the study protocol must also be provided in the manuscript.

Clinical data

Policy information about clinical studies

All manuscripts should comply with the ICMJE guidelines for publication of clinical research and a completed CONSORT checklist must be included with all submissions.

Clinical trial registration	ISRCTN45695543
Study protocol	https://pubmed.ncbi.nlm.nih.gov/31367635/
Data collection	We recruited participants from our local PR service. Patients were those referred to the service and HCPs were those who referred to the service from primary and secondary care sites.
Outcomes	Our research objectives were to understand:<br>• Patient and HCP perceptions of patients' decision-making needs using the current PR approach: How do they perceive this approach with regard to its barriers, facilitators, and improvements?<br>• Patient and HCP perceptions of patients' decision-making needs using a menu-based approach: How do they perceive this approach with regard to its barriers, facilitators, and improvements?<br>We conducted inductive data analysis using the Enhanced Critical Incident Technique.
